# The Student’s Manual of Venereal Diseases

**Published:** 1886-09

**Authors:** 


The Student's Manual of Venereal Diseases—Being a concise description of those affections and of their treatment. By Berkeley Hill, M. D., Professor of Clinical Surgery University College, London; Surgeon to University College and the Lock Hospitals, and Arthur Cooper, M. D., Surgeon to the Westminster General Dispensary; formerly House Surgeon to the Lock Hospital. Fourth edition. Revised. Philadelphia: P. Blakiston Son & Co. 1886.

We have read this little book with a good deal of interest and find much in it that is valuble. We consider it the most valuable of the manuals issued by this well-known house.

